# Improving Documentation of Physical Health Plans After Patient Visits From General Hospital

**DOI:** 10.1192/bjo.2025.10669

**Published:** 2025-06-20

**Authors:** Shehroz Shakeel

**Affiliations:** Coventry and Warwickshire Partnership Trust, Coventry, United Kingdom

## Abstract

**Aims:** Patients in acute psychiatric wards often require physical health assessments at associated medical hospitals. At Quinton Ward, Caludon Centre, we frequently transfer patients to University Hospital Coventry and Warwickshire (UHCW) for physical health concerns. However, their discharge plans are often not documented on CareNotes upon their return, leading to gaps in continuity of care. This audit aimed to assess whether discharge plans were documented and identify areas for improvement.

**Methods:** A retrospective audit was conducted on the last 10 patients transferred from Quinton Ward to UHCW for physical health concerns. Data collection focused on patient legal status, accompaniment by staff, presence of discharge documentation, and whether discharge plans were chased and recorded on CareNotes.

**Results:** 5 patients were informal, and 5 were detained under the Mental Health Act.

All 10 patients were accompanied by a staff member.

7 out of 10 visits were to A&E only, while 3 patients were admitted, including 2 readmissions.

Only 2 patients returned with a formal discharge summary.

No staff member actively chased a discharge summary or treatment plan for the remaining 8 patients.

Of the 2 patients with a discharge summary, only one had full documentation of their discharge plan on CareNotes, while the other had partial documentation.

Overall, only 1 out of 10 patients had a fully updated physical healthcare plan upon return.

No documentation was found regarding whether patients were satisfied with the care received at UHCW.

**Conclusion:** This audit highlighted a significant gap in the documentation of physical health treatment plans for psychiatric inpatients returning from UHCW. Given that discharge summaries are not always provided, relying on them is not a viable solution. To improve documentation, a structured form was developed for staff to complete while at UHCW or upon the patient’s return. This form ensures that essential information – diagnosis, investigations, treatment, and follow-up plans – is consistently recorded and uploaded to CareNotes. A follow-up audit will assess the effectiveness of this intervention in improving documentation and patient care continuity.

